# COVID-19 Highlights a Critical Need for Efficient Health Information Systems for Managing Epidemics of Emerging Infectious Diseases

**DOI:** 10.3389/fpubh.2021.767835

**Published:** 2021-12-10

**Authors:** Shalini Pooransingh, Raisa Abdullah, Stephanie Battersby, Ramoutar Kercheval

**Affiliations:** ^1^Public Health & Primary Care, Faculty of Medical Sciences, The University of the West Indies St. Augustine, St. Augustine, Trinidad and Tobago; ^2^Palliative Care Unit, Caura Hospital, Caura, Trinidad and Tobago; ^3^Department of Medicine, Eric Williams Medical Sciences Complex, Mount Hope, Trinidad and Tobago; ^4^Department of Surgery, San Fernando General Hospital, San Fernando, Trinidad and Tobago

**Keywords:** health information, surveillance, public health, communicable diseases, COVID-19, IHR (2005)

## Introduction

Health information systems are a fundamental component of effective Public Health programmes. They underpin effective and efficient surveillance and response systems. Health information systems comprise data and personnel. The data include health service data and health related data which together allow for the planning and evaluation of healthcare services. Health service data are generated from the moment a patient is seen by a doctor. In the case of infectious diseases the clinician notifies the disease to the public health authorities usually through passive surveillance or in the case of new serious disease through active or enhanced surveillance. Infectious diseases differ from non-communicable diseases in that the speed of response is key to limiting disease spread within the community. The data are sent to the institution responsible for collating and analyzing the data, usually an epidemiology unit. Health information systems are also critical to managing the data associated with contact tracing and follow up.

The purpose therefore of this paper is to demonstrate how the COVID-19 pandemic highlighted the valuable role of Health Information Systems in managing epidemics of emerging infectious diseases.

The International Health Regulations (IHR 2005) were revised in 2005 following global threats such as severe acute respiratory syndrome (SARS), avian influenza and the terrorist events of 2001 that the world faced in the preceding years (1). The regulations were revised to assist countries to better manage public health threats with a shift away from disease-based responses. The IHR 2005 sought to strengthen alert and response capacity in countries to minimize morbidity and mortality from infectious disease threats and threats due to non-infectious causes such as chemical and radionuclear hazards; and to minimize global spread of disease through early detection and reporting and timely sharing of information to relevant agencies (1).

## IHR 2005 Areas Of Work

The World Health Organization set out clearly the IHR 2005 areas of work (2) required to achieve the goal of early detection and response. Fundamental to this goal was ensuring surveillance and response capacities were strengthened. Countries have had from 2007 to plan and execute their preparedness plans to ensure capacities are in place to manage public health threats.

## Monitoring IHR 2005 Capacities

Every year countries complete States Parties reports on their progress with the implementation of the IHR 2005 requirements (3). These reports are based on self-assessment. Voluntary joint (country experts and an external team) external evaluations are carried out to assess the status of countries' preparedness to address public health risks (4). Some countries have had the opportunity to test their preparedness efforts in real events such as Ebola virus diseases, Middle East Respiratory Syndrome (MERS), and some countries learned from SARS prior to the revision of the IHR 2005 (5, 6). All countries' public health systems are being tested in the current COVID-19 pandemic.

## COVID-19 And Health Information

COVID-19 is an infectious disease spread through droplets and by aerosols (7). The initial responses in countries relied on active surveillance and effective and efficient contact tracing to minimize community spread. The World Health Statistic report 2021 noted a lack of disaggregated COVID-19 data and the absence of robust data infrastructure. According to the WHO Director General, “one of the greatest lessons from the pandemic is the importance of timely, reliable, actionable and disaggregated data…requires strong country data and health information systems through collaboration…” (8). The pandemic also demonstrated the critical role of health workers in public health, and demonstrated their inequitable availability.

## Discussion

The IHR 2005 are a useful framework for assisting countries in their planning and preparedness for public health events. The real test to determine if all preparedness efforts have resulted in a robust system is a real event such as the COVID-19 pandemic. COVID-19 has revealed that countries were not prepared for handling national outbreaks on the scale experienced with COVID-19. Under the IHR 2005, countries monitor their surveillance and response capacities through self-reporting mechanisms and through external evaluations. An independent review was undertaken to review the functioning of the IHR 2005 during COVID-19. The review found that countries were not prepared and suggested that perhaps the tools used to monitor countries' capacities may need revisiting (9).

Looking specifically at the Surveillance indicator in 2019 in the States Parties self-assessment tool for selected countries—Canada, China, Republic of Korea, and the United Kingdom (UK) scored 100%; while New Zealand scored 80% and Trinidad and Tobago 40% (10).

Some countries where the initial COVID-19 responses were noted as successful included China, New Zealand, and the Republic of Korea.

China had in existence a national online infectious disease reporting system implemented after SARS. This enables real-time gathering of case data. However, this system only applies to known diseases which medical practitioners and health facilities report using a reporting list. During COVID-19, China advanced isolation measures. All residents were required to go through comprehensive health assessments and obtain a code based on the green, yellow, and red traffic light system indicating no risk, moderate risk and high risk of COVID-19, respectively. COVID-19 led to innovation and the use of modern information technologies (11).

The Republic of Korea implemented a trace, test and treat strategy which they learnt from managing MERS in 2015 (5). They focussed on proactive case finding, contact tracing and isolation. The Korea Center for Disease Control (KCDC) can share data widely with relevant authorities such as central, municipal, or local governments, national health insurance agencies, and health care professionals. In March 2020 the KCDC launched the COVID-19 Epidemiological Survey Prompt Support System for enhanced contact tracing. This system enabled prompt delivery of data pertaining to infected individuals to epidemiology investigators immediately after requisite data were collected on a near real-time basis. This was possible as the legal and technical infrastructure were already in place (12).

New Zealand relied on their existing pandemic plan, which was based around influenza. New Zealand's success was attributed to its robust surveillance along with laboratory testing for SARS- CoV- 2. Identification of transmission chains early on along with the nationwide quarantine period helped to curb the number of COVID- 19 infections. Identification and containment of these transmission chains aided in protection of those most vulnerable within the population. In New Zealand, there is direct communication between the electronic health information systems at the primary care and hospital levels, which allows immediate access to relevant information such as referrals, laboratory results and discharge summaries (13).

Looking at other countries' preparedness in the area of surveillance and their actual response to the pandemic, the scores do not appear to match; there may be other factors at play.

The UK's initial response was described as too little, too late and not adequate despite the UK thinking it was prepared. In the UK, every indicator achieved 100% on the States Parties reporting tool for 2019; but they struggled with controlling their epidemic in the early phases.

A report on the lessons from COVID-19 described several key issues, however the focus for this paper is health information. The report noted “Data-sharing from organizations such as PHE (Public Health England) and Department of Health and Social Care (DHSC) has been wholly inadequate … the lack of data on testing … has left local areas with no mechanism for monitoring the number of confirmed cases of COVID-19… Local arrangements that have been put in place to try and address the gaps in national data-sharing have required lengthy manual data cleansing processes.” (14).

A good practice example was seen from the Greater Manchester Combined Authority (GMCA) which made innovative use of data during the pandemic. For example, 10 councils in the GMCA area created a digital dashboard where care homes reported issues such as COVID-19 outbreaks and shortages in personal protective equipment. The dashboard enabled other local health and social care providers to offer support (14).

Canada does not work operate a standardized system for surveillance; and data collection and analysis are not consistent across the country. Early in the COVID-19 pandemic, the federal government recommended that provinces and territories collect specific demographic and epidemiological data and report their findings to the Public Health Agency of Canada (PHAC). Although Canada scored 100% on the surveillance indicator on the State Parties tool, the lack of national standards can pose limitations in the effectiveness and efficiency of the surveillance system (15).

In contrast countries with less good surveillance indicators such as Trinidad and Tobago with a score of 40%, perhaps mindful of their health system weaknesses, implemented border closures early and non-essential services lockdowns; they fared well early on in the pandemic with low cases numbers. However, increasing case numbers added to a weak surveillance system and limitations in human resource capacity means the country continues to rely on lockdowns 18 months into the pandemic (16).

The speed at which decisions and actions are taken are critical in a communicable disease response. Additionally, the ability to scale up an already strong and robust system as in China and Republic of Korea for example, will produce better quicker results than trying to scale up a less robust system.

During the 74th World Health Assembly in May 2021, strengthening preparedness for and response to emergencies was deliberated upon, resulting in WHA resolution 74.7 which included urging Member states (17):

- “to strengthen their capacities, to strengthen their systems for surveillance required under the International Health Regulations (2005),
- to strengthen their core public health capacities and workforce for indicator-based and early-warning surveillance to ensure that all relevant events are rapidly detected and controlled;
- to work toward achieving strong and resilient health systems”

Although it is implied in the IHR document Annex 1 and in the IHR areas of work that countries need to have capacities for surveillance and response in place, judging from the responses and issues highlighted in countries, the requirements for effective surveillance may need to be detailed further. Parameters such as manual or electronic systems, national standards, local level systems communicating with the national level, and a trained workforce to support a surge may need to be included. Indeed, the WHO Benchmarks for IHR 2005 document (18) attempted to do this. This document, published in February 2019, perhaps did not allow countries the time to implement ahead of their 2019 State Parties report, and also ahead of the COVID-19 pandemic. Although countries could use this tool to improve their health information systems, some countries will require additional support to achieve these parameters. The less resourced countries will not have the trained human resource capacity and even the computer and internet connectivity requirements to implement the requirements.

Although countries with high scores on the IHR 2005 assessment tools may have thought they were prepared for a Public Health emergency, the scale of the pandemic was not anticipated and the pandemic highlighted several areas requiring attention. Countries with lower scores like Trinidad and Tobago should have perhaps been planning earlier to strengthen their surveillance and response systems i.e., stronger health information systems to track and trace along with a stronger public health workforce through the training of other healthcare workers and volunteers. Indeed, in the absence of a real-live event to highlight gaps, though labor-intensive, there is no escaping planning and preparedness which must include carrying out simulations and drills to test plans and procedures which will make it easier to function effectively and efficiently during a crisis (19, 20).

In conclusion, a strong health information system is paramount. It is vital in surveillance and response and for monitoring progress toward the sustainable development goals, global programme of work 13 Triple Billion targets, and national and subnational health priorities. An effective system will generate timely, reliable, disaggregated comparable and actionable data, to measure and track population health determinants and outcomes along with health inequalities data, shown to be essential in COVID-19 and which will drive strategic policy changes.

## Author Contributions

SP conceived the paper, did the research, and wrote the paper. RA, SB, and RK did research, read, and edited the draft. All authors contributed to the article and approved the submitted version.

## Conflict of Interest

The authors declare that the research was conducted in the absence of any commercial or financial relationships that could be construed as a potential conflict of interest.

## Publisher's Note

All claims expressed in this article are solely those of the authors and do not necessarily represent those of their affiliated organizations, or those of the publisher, the editors and the reviewers. Any product that may be evaluated in this article, or claim that may be made by its manufacturer, is not guaranteed or endorsed by the publisher.
